# Rapidly adapting an effective health promoting intervention for older adults—choose to move—for virtual delivery during the COVID-19 pandemic

**DOI:** 10.1186/s12889-022-13547-5

**Published:** 2022-06-11

**Authors:** Samantha M. Gray, Thea Franke, Joanie Sims-Gould, Heather A. McKay

**Affiliations:** University of British Columbia, Centre for Hip Health and Mobility, 2635 Laurel Street, Vancouver, BC V5Z 1M9 Canada

**Keywords:** Physical activity, Implementation science, Seniors, Adaptation

## Abstract

**Background:**

The COVID-19 (COVID) pandemic shifted way of life for all Canadians. ‘Stay-at-home’ public health directives counter transmission of COVID but may cause, or exacerbate, older adults’ physical and social health challenges. To counter unintentional consequences of these directives, we rapidly adapted an effective health promoting intervention for older adults—Choose to Move (CTM)—to be delivered virtually throughout British Columbia (BC). Our specific objectives were to 1. describe factors that influence whether implementation of CTM virtually was acceptable, and feasible to deliver, and 2. assess whether virtual delivery retained fidelity to CTM’s core components.

**Methods:**

We conducted a 3-month rapid adaptation feasibility study to evaluate the implementation of CTM, virtually. Our evaluation targeted two levels of implementation within a larger socioeconomic continuum: 1. the prevention delivery system, and 2. older adult participants. We implemented 33 programs via Zoom during BC’s 1st wave acute and transition stages of COVID (April–October 2020). We conducted semi-structured 30-45 min telephone focus groups with 9 activity coaches (who delivered CTM), and semi-structured 30-45 min telephone interviews with 30 older adult participants, at 0- and 3-months. We used deductive framework analysis for all qualitative data to identify themes.

**Results:**

Activity coaches and older adults identified three key factors that influenced acceptability (a safe and supportive space to socially connect, the technological gateway, and the role of the central support unit) and two key factors that influenced feasibility (a virtual challenge worth taking on and CTM flexibility) of delivering CTM virtually. Activity coaches also reported adapting CTM during implementation; adaptations comprised two broad categories (time allocation and physical activity levels).

**Conclusion:**

It was feasible and acceptable to deliver CTM virtually. Programs such as CTM have potential to mitigate the unintended consequences of public health orders during COVID associated with reduced physical activity, social isolation, and loneliness. Adaptation and implementation strategies must be informed by community delivery partners and older adults themselves. Pragmatic, virtual health promoting interventions that can be adapted as contexts rapidly shift may forevermore be an essential part of our changing world.

## Background

### The epidemic of loneliness, social isolation and physical inactivity

Loneliness and social isolation were declared a global epidemic by former U.S. Surgeon General Vivek [1], affecting one-third or more of older adults [2]. Issues of loneliness and social isolation are often overlooked by health and social service professionals although they require clinical and social interventions. The long-term (greater than 4 years) effects of both can be devastating—they include increased blood pressure, depression, anxiety, weight gain, smoking and alcohol/drug use, stroke, coronary heart disease and alone time [3, 4], and accelerated loss of physical functioning and health with age [5]. Loneliness also predicts reduced levels of physical activity [6, 7].

Physical activity is a modifiable lifestyle factor that effectively prevents and manages a host of chronic diseases [8], which in turn curbs escalating health care costs [9]. Physical activity reduces the risk of all-cause mortality, cardiovascular disease, falls, anxiety, and depression, preserves older adults’ mobility and independence [8]. However, older adults comprise the least physically active segment of the population in many developed countries [10–13]. Eighty-seven percent of older Canadians do not meet guidelines of 150 min/week of moderate-to-vigorous intensity physical activity [14].

### The COVID-19 pandemic

As COVID-19 (COVID) spread across the globe, our way of life shifted. On March 11, 2020 the World Health Organization declared COVID a pandemic [15]. Five days later British Columbians in Canada came under strict physical distancing orders from Public Health Offices [16]. Large gatherings were banned and most indoor public spaces were closed, including recreation facilities [17]. While COVID posed a risk to all Canadians, older adults were most at risk for COVID and faced more serious illness and death than any other population group [18–21]. Public health guidelines that directed older adults to stay-at-home and sustain physical distancing protocols laid bare an escalating trend toward increased social isolation and loneliness among older adults. Both have been strongly linked to poor mental health [22] and quality of life [23] during the COVID pandemic.

In Canada, and elsewhere, stay-at-home orders slowed viral spread, during a time when no preventive pharmacological approaches or treatments existed for COVID [24]. However, these orders reduced older adults’ physical activity levels [25, 26]. During France’s 55-day national confinement period in the spring of 2020, 39% of 1178 older adults reported reduced levels of physical activity [27, 28]. Physical activity decreased more among older adults who lived in urban areas (43%), compared with those living in rural areas (32%; *p* < 0.001) [27]. In Japan, there was a significant decrease (*p* < 0.001) in total older adult physical activity time during the first lockdown (April 2020), compared with January 2020 activity levels [29]. In Brazil, there was a significant decrease in daily steps (β = − 886 steps/day, *p* = 0.018) and moderate-to-vigorous physical activity (β = − 2.8 min/day, p = 0.018), as well as a trend toward decreased light physical activity (β = − 26.6 min/day, *p* = 0.053) [30] among older adults before (January–March 2020) and during (June 2020) the pandemic.

### Countering the consequences of COVID through physical activity

Safe, well-designed countermeasures are one means to prevent unintended consequences of COVID public health directives [31, 32]. Interventions designed to increase long-term physical activity participation and promote social connectedness [33] may effectively stop or slow mobility loss [34] and diminish loneliness [35–37]. Thus, physical activity is a viable strategy by which older adults can maintain their overall social, mental [38], and physical health status and lower their risk of contracting COVID [39, 40].

Choose to Move (CTM; described in Methods) is an effective health promoting intervention that was scaled up in phases (2016–21) across British Columbia (BC), engaging > 2000 older adult participants. CTM effectively enhanced social connectedness, mobility, and physical activity and reduced loneliness [41, 42]. Prior to COVID, CTM was an in-person program. With community partners, our Active Aging Research Team (AART; https://activeagingrt.ca/) rapidly adapted CTM in March 2020 so it could be delivered to older adults virtually during the pandemic.

### Guiding frameworks

Implementation, scale-up and evaluation of CTM were guided by Yamey’s scale-up Framework for Success [43] and the Framework for Successful Implementation [44] that embeds elements of the Interactive Systems Framework (ISF) [45]. The ISF [45] references six categories of factors that influence effective implementation; the *innovation*, the *prevention delivery system* (e.g., individuals, organizations, or communities that deliver the intervention), the *prevention support system* (e.g., central training and assistance), and the *prevention synthesis and translation (research) system*–embedded within a broader socioecological context defined by *provider and community characteristics* [44, 45]. Ongoing development, adaptation, and evaluation in diverse contexts and populations across systems as per the ISF, are necessary to achieve participant health impact [46, 47].

### Aim

Therefore, our overall aim was to counter unintended consequences of the public health ‘stay-at-home’ directives on older adults’ health. To do so we rapidly adapted CTM with our community partners, to deliver CTM virtually.

### Objectives

Our specific objectives were to: 1. describe factors that influence whether virtual delivery of CTM was acceptable and feasible to deliver, and 2. assess whether virtual delivery retained fidelity to CTM’s core components.

## Methods

### Context

#### Choose to move

In 2015, with funding from BC Ministry of Health, we collaborated with community partners to co-create a flexible, effective [41], community-based health promotion intervention called CTM (https://www.choosetomove.ca). We described the components and implementation of CTM in great detail elsewhere [41, 48, 49]. Briefly, CTM was a 6-month, choice-based physical activity and social connectedness model that supports older adults to become more physically and socially active through three components: (1) a one-on-one consultation with an activity coach (AC), (2) regular ‘check-ins’ with the AC, and (3) motivational group meetings with other CTM participants (up to 12 participants/group). CTM is flexible in that participants create an action plan that suits their interests, abilities, income, and available resources. In group sessions, participants share their experiences and challenges executing their action plan and connect with others. During small scale delivery (2016–2017; *n* = 458 older adults), physical activity increased significantly during the first 3 months of CTM (baseline-3 months) in younger (60–74 yrs.; + 1.6 d/wk.; *p* < 0.001) and older (≥75 yrs.; + 1.0 d/wk.; *p* < 0.001) participants. The increase was sustained at 6 months (post-intervention) in younger participants only, who remained significantly more active than at baseline (+ 1.4 d/wk.; *p* < 0.001). Social connectedness scores improved significantly in the younger group at 3 (﻿*p* < 0.001) and 6 months (﻿*p* = 0.02) [41]. CTM aligned with delivery partner organizational priorities, visions, and strategic directions and was deemed feasible and acceptable to deliver [49].

Prior to the COVID pandemic, we scaled up CTM in BC across three phases. In Phases 1, 2 (2016–2017) and 3 (2018–2020), 2128 older adults participated across 99 BC communities (urban and rural). We formally adapted CTM based on delivery partner and participant feedback at every phase [50]. For Phase 4 we adapted CTM to increase reach and reduce implementation costs, while maintaining fidelity to CTM core components [described below]. Before we could roll-out Phase 4 programs (March 2020) strict public health orders, due to COVID, were enacted.

#### Rapid adaptation of CTM for virtual delivery

In March 2020, we engaged longstanding community delivery partners to adapt CTM so that it could be delivered virtually. Our goal was to rapidly address escalating levels of social isolation, loneliness, and physical inactivity among older adults during the pandemic. CTM became a 3-month (rather than 6-month) intervention with six virtual group meetings delivered by ACs every 2 weeks. We retained participant’s one-on-one consultations with ACs and virtual group meetings (six × 1 hour), as we considered them ‘core’ components to foster social connections and support participants’ physical activity goals. *Core components* are fundamental aspects of the intervention [51], considered potential drivers of health impact. About 80% of older adults could access online group meetings; however, we also provided a phone-in option to increase access to CTM group sessions. ACs provided support to participants to help them become acquainted with the technological platform for group meetings (e.g., support to use Zoom). We adapted the AC training module to reflect adapted CTM content, populated the CTM website with reputable resources, and distributed a virtual newsletter to older adults to support them to be active at home.

### Study design

We conducted a 3-month rapid adaptation feasibility study. Across levels of influence, we focused on; 1. the prevention delivery system [45], and 2. older adult participants, to evaluate implementation of CTM virtually.

### Choose to move for virtual delivery—timeline

Virtual delivery of CTM spanned COVID’s 1st wave in BC [acute and transition stages], as per the province’s Restart Plan (www.gov.bc.ca/restartbc). The COVID acute stage included CTM programs with start dates between April 20 and May 18, 2020 (20 programs). The COVID transition stage included programs with start dates between May 19 and August 6, 2020 (13 programs) (see Table 1). A similar proportion of older adults participated during the COVID acute stage (48%) as during the transition stage (52%).

**Table 1:** Virtual delivery of Choose to Move in 14 sites with a total of 33 programs

Delivery partner	Site (Group)	COVID Phase	Invited to participate	Agreed to participate	Consented to evaluation
YMCA	Chilliwack	Acute	35	10	8
BCRPA	Coquitlam - Dogwood	Acute	11	8	3
BCRPA	Coquitlam - Glen Pine Pavilion 1	Acute	13	10	4
BCRPA	Coquitlam - Glen Pine Pavilion 2	Acute	9	7	3
BCRPA	Coquitlam - Glen Pine Pavilion 3	Acute	12	8	4
BCRPA	Cranbrook	Transition	10	6	2
YMCA	Eagle Creek	Transition	35	8	6
YMCA	Kamloops John Tod Centre Y	Transition	20	6	6
YMCA	Kelowna Family	Transition	20	9	5
BCRPA	Kent/Agassiz	Acute	15	9	6
BCRPA	Langley	Acute	24	11	5
BCRPA	Maple Ridge	Transition	22	11	11
BCRPA	Mission	Transition	25	7	6
BCRPA	Mixed site: Surrey, Quesnel, Victoria, Smithers	Transition	9	9	5
BCRPA	Mixed site: Burnaby - Bonsor 55+, Burnaby - Confederation, Vancouver - Killarney	Transition	36	6	6
YMCA	Mixed site: Langara YMCA, Robert Lee YMCA	Acute	40	10	6
BCRPA	Mixed site: Langley, New West, Newton	Transition	60	11	7
BCRPA	Mixed site: Port Moody, Maple Ridge	Transition	15	7	4
YMCA	Mixed: Eagle Creek, Robert Lee, Tong Louie, Chilliwack Y, Abbotsford Y, Guildford	Transition	15	11	10
BCRPA	New Westminster	Acute	18	11	7
BCRPA	Pitt Meadows	Transition	9	6	4
BCRPA	Prince George	Acute	12	5	2
YMCA	Prince George	Acute	18	8	4
BCRPA	Salmon Arm 1	Acute	8	6	0
BCRPA	Salmon Arm 2	Acute	7	6	2
BCRPA	Surrey - Guildford	Transition	15	12	7
BCRPA	Surrey - Newton	Acute	18	8	6
BCRPA	Trail 1	Acute	9	5	1
BCRPA	Trail 2	Acute	8	4	0
BCRPA	Vernon	Acute	21	6	5
BCRPA	West Kelowna 1	Acute	10	8	4
BCRPA	West Kelowna 2	Acute	8	6	3
BCRPA	West Kelowna 3	Acute	8	7	1
		**Totals**	595	262	153

### Interactive CTM systems

#### Prevention delivery system (delivery team)

The CTM Prevention Delivery System was comprised of three groups. First, the leads of two delivery partner organizations had capacity to work with us to adapt and deliver CTM virtually during COVID. Second, recreation managers coordinated hiring the ACs, and third, ACs who delivered CTM and were in direct contact with older adult participants.

#### Prevention support system (central support unit)

Members of our team (AART) at the University of British Columbia comprised the prevention support system (we use the term central support unit throughout). They worked with delivery partners to gauge capacity of the organizations to deliver CTM (e.g., time, staff, computers), to facilitate all aspects of CTM planning, design, and implementation, to design, adapt, and deliver the AC training module, and to adapt (with delivery partners) CTM for virtual delivery. The central support unit provided technical assistance specifically to address problems and/or queries of delivery partners [52]. Support came in the form of phone and email check-ins with delivery partners, ongoing AC training support (including Zoom technology support), and provision of materials and resources to ACs to deliver CTM virtually.

#### Prevention synthesis and translation system (research team)

Members of our team also comprised the Prevention Synthesis and Translation System (research team) [45]. The research team’s primary role was to evaluate implementation and effectiveness of CTM and to distribute information and outcomes to participant, community and government stakeholders [45].

### Implementation evaluation indicators

As per our guiding frameworks, we acknowledge that different factors influence implementation of CTM along a socio-ecological continuum that spans community level factors, provider characteristics, and intervention (CTM) characteristics. We focus specifically on acceptability, feasibility, fidelity, adaptation, and dosage (dose delivered) [53] of CTM at the level of the AC (see Table 2). We focus here as our previous studies highlight the essential role of ACs to implementation success [42, 50].

**Table 2:** Key terminology

**Acceptability** (determinant): perceptions among the delivery team that a given intervention is agreeable, palatable, or satisfactory [54]	
**Adaptation** (outcome): planned or purposeful changes to the design or delivery of an intervention; can also include unintentional deviations from the intervention as originally designed [55]	
**Dose delivered** (outcome): intended units of each intervention component delivered to participants by the delivery team [56]	
**Feasibility** (determinant): perceptions among the delivery team that an intervention can be successfully used or carried out within a given organization or setting [54]	
**Fidelity** (outcome): the extent to which an intervention is implemented as it was prescribed in the intervention protocol – by the delivery team [57]	

We report outcomes and determinants, defined below, that were recommended as part of a minimum data set of implementation indicators deemed most relevant for the implementation of physical activity and behavioural nutrition interventions [53]. Outcomes refer to the effects of deliberate actions to implement an intervention (e.g., fidelity, adaptation, dose delivered) [54]. Determinants refer to the range of contextual factors that influence implementation (e.g., acceptability, feasibility) [44]. Further, we assess barriers and facilitators to virtual implementation of CTM.

### Data collection

#### Activity coaches

All (*n* = 15) ACs were invited to participate in focus groups. We conducted three focus groups with ACs that consented to participate (*n* = 9) over videoconference on Zoom (July 2020; September 2020) after they completed virtual delivery of CTM. The purpose was to assess the AC experience delivering CTM virtually. Questions focused on: acceptability and feasibility; barriers and facilitators to delivery; and adaptations made during delivery. We used purposeful sampling [58] and separated ACs into focus groups so we had representation from different BC health authorities to ensure that different delivery partners, and urban and small urban communities were represented.

#### Older adults

As shown in Table 1, there were 153 older adults enrolled in CTM virtual programs who consented to be evaluated. From among them, we invited 32 older adults who participated in CTM programs during acute and transition stages of the 1st wave of COVID to partake in interviews at baseline and program completion (3-months). We used purposeful sampling [58] to select CTM participants from across all five health BC health authorities to ensure representation across sex, age groups, varying delivery partners, and from urban and small urban locations (see Table 1).

We conducted 15 telephone interviews, of approximately 30–40 minutes duration, with older adults during COVID acute and transition stages (*n* = 30); five interviews were conducted at baseline and 10 at program completion (3-months) for each stage. Please see Table 3 for a demographic summary of older adult participants who were interviewed. The purpose of these interviews was to obtain feedback from older adults who were participating in CTM virtually, to examine whether the program facilitated their physical activities and social connections during COVID (Table 4).

**Table 3:** Demographic summary of older adult participants who were interviewed

Stage	Time Point	Sex	Ethnicity	Age Range
Acute (*n* = 15)	Baseline (*n* = 5)	Men (3/5) Women (2/5)	White (3/5) Black (1/5) South Asian (1/5)	71–82 years
	3-months (*n* = 10)	Men (2/10) Women (8/10)	White (7/10) Chinese (1/10) Southeast Asian (1/10) South Asian (1/10)	66–89 years
Transition (*n* = 15)	Baseline (*n* = 5)	Men (2/5) Women (3/5)	White (4/5) Filipino (1/5)	70–79 years
	3-months (*n* = 10)	Men (3/10) Women (7/10)	White (10/10)	67–84 years

**Table 4:** Example older adult interview questions

**What options were or were not open to them at the time they enrolled.**	
1. To begin, I was hoping you could share with me why you decided to participate in Choose to Move.	
**How has COVID-19 influenced their behaviour.**	
*Socialization and COVID19*	
1. Can you share what your social interactions and relationships looked like before starting Choose to Move?	
2. Currently, what is your biggest challenge to maintaining your meaningful relationships? So those closest to you that you care about or those that care about you?	
3. What are your ideas on ways to improve your meaningful relationships while remaining at home?	
*Physical Activity/Mobility and COVID19*	
1. How have social/physical distancing measures impacted your physical activities?	
2. Currently, what is your biggest challenge to maintain regular physical activity?	
**Access to resources – how does this vary based on geography and how does it influence how older adults are affected by COVID-19 and their participation in CTM**	
**Equity, Community and Online Resources**	
1. How has COVID-19 impacted your awareness about the resources and opportunities available to you in the community and online?	
**CTM recommendations**	
1. What could we add to CTM to make it more beneficial for you?	

### Analysis

#### Qualitative

All interviews and focus groups were audio-recorded and transcribed verbatim by a professional transcriptionist. Data were de-identified and imported into NVivo 11 for data analysis. We reviewed transcripts using a deductive framework analysis; framework analysis is well suited to research that has specific questions, a pre-designed sample, and a priori issues [59]. In deductive framework analysis, the categories/codes are often pre-defined (e.g., by specific areas of interest to the project). AC focus groups were coded to capture factors that influenced the acceptability and feasibility of delivering CTM virtually, and adaptations made during delivery of CTM and the relationship between changes and fidelity to the core components. Older adult interviews were similarly coded. We integrated storylines from older adult interviews with AC focus group data to evaluate and/or track major facilitators and barriers to achieving implementation goals [60] (delivery and impact).

There are seven stages to framework analysis [59, 61, 62]. We briefly describe how we enacted each stage, below. First, the lead author received the transcripts (stage 1 - transcribe), read through the transcripts to become more familiar with the interviews (stage 2 - familiarize). Although we had pre-defined categories/codes we conducted open coding on two transcripts to ensure codes were not missed (stage 3 - code). We held a series of team meetings to discuss the framework (stage 4 - develop a framework). SMG and TF divided the remainder of the transcripts between them and coded the transcripts using the framework and added codes that were missing from the framework (stage 5 - apply the framework). We coded full paragraphs to retain contextual meaning [63]. We adopted the constant comparison method [64] to identify patterns and connections within and between cases and codes. This revealed similarities and differences in the data (stage 6 - chart). We then interpreted data by mapping connections between codes, to explore relationships and develop themes within each category (stage 7 - map). We used pseudonyms for participant quotations presented in the results.

## Results

### Acceptability and feasibility of delivering CTM virtually

ACs and older adults identified a number of factors that influenced the acceptability and feasibility of delivering CTM virtually. We present influencing factors under each implementation indicator. Factors that influenced acceptability were: (1) a safe and supportive space to socially connect, (2) the technological gateway, and (3) the role of the central support unit. Factors that influenced feasibility were: (1) a virtual challenge worth taking on and (2) CTM flexibility. We include a thematic summary table (see Table 5).

**Table 5:** Summary of themes and influencing factors

Theme	Influencing factors
Acceptability	A safe and supportive space to socially connect The technological gateway The role of the central support unit
Feasibility	A virtual challenge worth taking on CTM flexibility
Fidelity	Time allocation (content, contact time) Physical activity levels

#### Acceptability

##### Acceptability factor 1: a safe & supportive Space to socially connect

ACs highlighted CTM as a welcome opportunity for many older adults during a time when health orders mandated shut-downs and people were required to stay at home. Through CTM, ACs felt they created a safe space for older adults to come together, find support, and remain accountable to their physical activity action plans. The virtual space was COVID-safe—no risk of exposure or transmission—and emotionally-safe as ACs welcomed older adults to share their stories and struggles without judgement. Through CTM, ACs provided older adults timely support to stay mobile in their homes.

To start setting some goals and have some accountability so that they got more active … Many of them definitely felt they had decreased their activity levels as well as their social connectedness since COVID. So, everyone was really happy to have a chance to come back together with likeminded individuals who share common goals and share experiences with each other. – Lianne, AC [transition]

I was happy. I got calls, we call, you know. I had connections still with people through the phone, email. – Female, 78, Filipino [transition]

Participants within groups also upheld a safe and welcoming environment. Participants showed one another kindness, the opportunity to share experiences, and support one another.

What was so lovely about that is the one guy who has these challenges, the kindness in the group, even when we’re on Choose to Move, kindness meaning the respect to let the other person talk, to let them tell their story, to let them travel through the struggle, you know, and a lot of people were struggling. – Lilian, AC [acute]

I had-- well, all along I had lost my wife, so my social aspect had deteriorated until I got involved [in CTM]. – Male, 82, white [acute]

##### Acceptability factor 2: the technological gateway

While CTM was largely perceived as agreeable, it was not fully satisfactory in all cases. Technology inevitably presented challenges for both ACs and participants.

Well, to begin with I am not very adept at technology. It takes a while to get my confidence up. And I had to get a lot of help which was good. Because the person who has been leading the session, is very patient and forgiving. Female, 80, black [acute]

ACs felt challenged to build connections with participants, and foster connections among participants in the virtual setting. ACs noted that the virtual setting was less conducive than in-person settings to relationship-building, especially when ACs could not see participants on video.

Challenges for me was not being able to see my participants. I found that really difficult. I really like to … kind of watch how they move and what that looks like. I felt like I just wasn’t connecting as well as I would have wanted to with them. And it felt like they didn’t get that connection with me. – Katie, AC [acute]

ACs remarked that older adult participants also experienced technological challenges—some did not feel comfortable connecting to online platforms for group meetings. Others lacked trust in the platforms, citing concerns that their computer or device might get a virus. This meant that some participants called-in only (no video). As they listened and chose to remain muted, they were unable to speak or contribute to group discussions.

Another challenge was, of course, just getting people to buy in to going on Zoom or the phone call. A lot of the older adult population, unless they learn from their family or work or something, this kind of technology is kind of scary. That’s the feedback that I’ve got from them. … my computer it might get a virus from this and stuff like that. – Katie, AC [acute]

A few ACs mitigated technological issues by offering in-person, distanced outdoor meetings. The adapted in person delivery became acceptable to older adult participants.

There was one fellow that wouldn’t do the online but as soon as we went outside, he showed up to both meetings because he would say he was going to do the computer and he finally called me and said I just don’t want to do it. I said that’s fine. You know what, nobody says you have to and that’s all good. We’ll figure out something else. Maybe I’ll meet you in a park and we’ll have a coffee together. But he showed up for the last two and he loved it. – Lilian, AC [acute]

##### Acceptability factor 3: the role of the central support unit

The acceptability of CTM for ACs relied in part on resources and supports, that the central support unit put in place. Ideas for ice-breaker activities in the group meetings and technical support were central support unit services that elicited ‘satisfaction’ from ACs.

Something that worked really well were the icebreakers. Because even though one of the groups I was running, a lot of them knew each other. They hadn’t seen each other for a long time. So those icebreakers were really fun. – Emma, AC [acute, transition]

ACs felt that the central support unit provided them adequate and up-to-date resources to deliver CTM virtually, and to support their participants to engage fully in CTM. For some ACs, the resources formed a jumping-off point to seek out additional resources, in response to participant needs.

All of the resources were there. And I always went through them, like, a week before, just to make sure I wasn’t missing anything. And then sometimes it would tweak me, and I would maybe check another resource ‘cause it may be provided some additional information. But I think kind of the meat and potatoes of what we needed was there. And the COVID information was good. It was nice to have that up-to-date information, because I think a lot of them were still maybe confused and had questions about COVID and maybe what phone numbers or websites to access. And I think there was also some information about mental health and the help line or the phone number that they could contact. And a lot of them actually found that information useful. – Priscilla, AC [transition]

#### Feasibility

##### Feasibility factor 1: a virtual challenge worth taking on

Connecting to a virtual platform presented technological challenges for participants and ACs. However, ACs felt that CTM was feasible to deliver virtually. ACs stated their commitment to mitigate technological concerns or issues by checking in on their participants (e.g., through a phone call or email). ACs showed great care, concern, and support for participants—even those who did not enroll in the intervention or who were ‘no-shows’.

The emails and the phone calls and then the day-of and then the reminder and there was a lot of that. And even sometimes you still didn’t get people on the call and you’re, like, what did I do wrong? Why are you not joining? … I think about a lot, like, sitting at home in this apartment, not doing anything. Are you even more sad that you’re not on?... Or a little lonelier or you just are not in the mood for it today because you’re too lonely. – Katie, AC [acute]

ACs firmly believed that the technological difficulties were worth ‘muddling’ through to achieve those connections and sustain contact with others while isolated during the pandemic in BC. They felt that what could be gained from attending the virtual group meetings—by phone or video—was much better than the alternative of no contact whatsoever.

I felt pretty comfortable with the technology. It’s always better to have people in the same room as you where you can get better cues off of their body language and such. But people learned to get pretty vocal and get beyond that little, tiny picture on the screen of somebody … Everyone was able to get on to video. Not always able to stay there... there was always somebody that had a difficulty in any given meeting … But I think that’s to be expected with a technology that most people weren’t familiar with. And considering the alternative, of shutting things down and not having that ability to interact and see those little faces, so much better than doing it by phone or just by email. – Ivy, AC [acute]

The technical glitches were a little bit of a stumbling block in the beginning, I have to admit. And not everybody has a computer. So we had-- one individual was on the telephone. And, you know, technical glitches aside, it’s good to see people’s faces – Male, 68, white [acute]

While technology use for the virtual group meetings could be a barrier, it was also viewed as a flexible means of attending sessions. Scheduling meetings was much easier to coordinate during periods of provincial lockdown because everyone had more time. Participants could choose to be on video or on the phone. They could also attend sessions in the safety and comfort of their homes, even if they did not feel well on a particular day.

It was easy to find a time that worked for everyone at home … And everyone has kind of a-- more of an empty calendar these days. I found that most people were pretty good at being able to attend the meetings whereas when we did them in person … there were lots of obstacles that prevented people from attending sometimes. I think the virtual aspect of it really does make it easier. Even if someone doesn’t feel that well that day, they’re still in the comfort of their own home and able to, you know, not have to travel anywhere to get to where they’re going. I do love the virtual aspect. But I think it’s probably not for everyone. – Lianne, AC [transition]

##### Feasibility factor 2: CTM flexibility

From its inception, CTM was designed to be flexible, not prescriptive. ACs took advantage of the flexible nature of CTM as it enhanced their ability to effectively deliver CTM in the COVID context. A flexible approach supported ACs to deliver CTM virtually, through a variety of modes (e.g., Zoom, phone, email) to be as inclusive as possible for all group members. This meant being creative and finding ways to support participants who did not have online video access or email.

I did really try and follow the script, and I didn’t necessarily bring my PowerPoints up onto the screen that my participants could see. Because some were on the phone, and some were via video. So, I always had my secondary laptop set up, and I would use it that way. I think that that was probably more fair too to the groups because some couldn’t see it and some could. – Emma, AC [acute, transition]

Below, Natalie describes how she was able to offer participants a chance to connect in person. Distanced meet ups better supported the needs of all participants, specifically those without virtual access.

I’d rely on the email to kind of send that out, and which is appropriate anyway ‘cause quite often it’s a link. That certainly didn’t help with the one who’s only on the phone. She has no P.C., no email... Actually, to get around that we did meet twice to walk as a group. So, she was able to see everyone … It gave them a chance to have some visual connection, which I think really helped. – Natalie, AC [acute]

### Fidelity to core components of CTM

We established that one-on-one consultations and group sessions were core to CTM effectiveness [41]. ACs made adaptations to CTM during implementation that comprised two broad categories: **time allocation** and **physical activity levels**. We describe the perceived influence of these adaptations on fidelity (fidelity-consistent, fidelity-inconsistent, or unknown) [65]. By fidelity consistent we mean adaptations that retain core components of the intervention [65]. Fidelity inconsistent adaptations refer to changes to CTM that altered core components [65], and potentially the intervention’s effectiveness.

#### Time allocation

ACs adapted how time was allocated during the implementation of CTM in response to participant needs.

##### Content

Tailoring CTM content within an individual meeting departed somewhat from the recommended time allocations for CTM group meeting activities, ACs did not interrupt or prolong discussion unnecessarily, and allowed participants to dictate the pace of the meeting. An AC described listening to participant needs in the context of group meeting content. We considered this adaptation to be fidelity-consistent.

We always did an icebreaker or discussion. But not always-- we didn’t use as much time as was suggested, the 30-minute timeframe. Mostly because, … I think they wanted to get into the meat of the meeting … we shifted the time more into the content discussion. – Ivy, AC [acute]

##### Contact time

ACs adapted CTM by adding, extending, or condensing their contact time with participants, all of which altered the dose delivered (amount and/or frequency; fidelity inconsistent). For example, some ACs added contact time and sent follow-up emails to provide resources or content from the meetings that were not delivered within the scheduled time. Others placed phone calls to check-in on participants

I did follow-up emails usually on the week between. And again, reminding them about the group challenge, any interesting article or link that might have come up. So just to kind of keep that contact and then a reminder of the next session’s meeting. – Natalie, AC [acute]

Sometimes, added contact time was to support participants through personal challenges or circumstances; informal listening/counselling that fell outside the core purpose of CTM (fidelity-inconsistent).

People would call me with personal stuff... She’s on, like, she needs someone to talk to. I’m on there for an hour. I’m going, sure, I’m not going to hang up on her, you know. – Lilian, AC [acute]

Two ACs held group meetings that ran longer than 1 h (the CTM guideline) which increased the contact time with participants (dose delivered; fidelity inconsistent).

You want to build that connection and sense of community. You can’t rush it. Either I had to skip over some of the presentations sometimes, shorten that piece, distribute some of that information and resources via email rather than discuss them in the meeting... we just talked about it and agreed that we would take up to an hour and a half to do the meetings, and we just did it that way. – Ivy, AC [acute]

CTM comprises 6 biweekly group meetings across 3 months. However, two ACs held their 6 meetings on a (mostly) weekly basis, thereby altering the frequency of dose delivered (but not the amount; fidelity inconsistent). ACs reported that many participants felt that weekly meetings prompted them to become more motivated to get active and revisit their goals.

We also had our meetings weekly instead of biweekly, and that was requested by the participants … And I personally felt, and I think all of the participants felt, that it was more motivating and encouraging … to literally see each other on a weekly basis... It really brought up their spirits, because we were also isolated for that two months where everybody was in lockdown. – Priscilla, AC [transition]

In summary, ACs described that the adaptations they made to **time allocation** were to balance a broad range of participant needs (e.g., social support and connection) while ensuring that CTM content was delivered as planned. Adding, extending, or condensing contact time with participants altered the dose delivered (amount and frequency) to participants. Therefore, we considered these adaptations fidelity-inconsistent as dose delivered and received could potentially influence participant level health outcomes.

### Physical activity levels

ACs adapted CTM to increase the level of physical activity during program contact time. Physical activity levels were adapted, in two ways: 1) through a change in setting (fidelity-consistent) and 2) by adding activities (fidelity-inconsistent).

For example, one AC conducted all group meetings outdoors during the transition stage of COVID in BC. This decision was made prior to the launch of her program by surveying interest among participants (fidelity-consistent).

They would bring their own chairs. They would wear masks if they wanted to. At the end of the day pretty much all but two people were totally thrilled about meeting at the park and being in the outdoors … It really brought up their spirits, because we were also isolated for that two months where everybody was in lockdown. And I think a lot of people were getting that Zoom fatigue. – Priscilla, AC [transition]

As another example, one AC introduced the option of planned group walks in addition to group meetings (fidelity-inconsistent). She described that the focus in her group shifted away from social aspects toward physical aspects of CTM through group walks: “*Certainly as we got into the later sessions, the social part didn’t seem to be quite as critical to them*” (AC). Added walks provided an opportunity to help participants find good, safe walking routes—something that ACs would usually provide in CTM through physical activity action planning.

This was additional, just to encourage them to find places they could walk … just to show them some of the different venues in town that they-- tracks basically or safe areas to walk. Plus, they were interested in how to use the walking poles. So, it was just kind of a little add-on we did. – Natalie, AC [acute]

Finally, another AC provided exercise class guidance to one of her CTM groups (fidelity-inconsistent).

And in the meantime, they actually wanted to start exercising … So, I got them going. I did a freebie one in the backyard. Got them going, … . -- they’ve been exercising every week. – Lilian, AC [acute]

In sum, adaptations in **physical activity levels** were a result of ACs finding ways to offer or integrate physical activity opportunities for their older adult participants—often in response to requests made by their participants—in CTM. A change in delivery setting was considered fidelity-consistent as ACs retained the type and amount of CTM core components. However, we considered cases where dose delivered increased (e.g., adding group walks, adding backyard exercise) as fidelity-inconsistent, given the potential for these changes to alter participant level health outcomes.

## Discussion

### Navigating the COVID pandemic

While epidemiologists warned for years of an impending pandemic, COVID caught most of the world off guard [66, 67]. Globally, response actions and response timelines to the COVID pandemic varied. In Canada, while efforts were made to protect our most vulnerable citizens, older adults were most at risk for COVID and faced more serious illness and death than any other population groups [20, 21]. Community dwelling older adults were homebound and in need of alternative physical activity and social opportunities to maintain their health during this time [68]. Our team sought to follow and act on the call for ‘research action at a distance’ [69] to support older adults in BC. We aimed to counter the unintended consequences of the stay-at-home directives on older adults’ health by adapting CTM for virtual delivery. By doing so, we extend the literature on the feasibility of adapting to online and remote delivery of health interventions for older adults—a population whose internet and technology use is typically below that of other population segments [70]. Below, we discuss the findings of our implementation evaluation in the context of the current literature.

### Implementation evaluation

Not unlike other sectors, our delivery team was confronted with myriad challenges (e.g., hiring staff, learning new technology, recruiting participants to an online program) while implementing CTM virtually during the pandemic. These challenges and their solutions are similar to other studies that sought to deliver virtual programs during COVID [71–73].

Two interrelated elements that surfaced within our implementation evaluation (technological challenges and AC adaptations) influenced implementation of CTM virtually; findings align with the broader literature.

#### Technological challenges

ACs received the support and training they required to deliver CTM virtually during the acute and transition stages of the 1st wave of COVID in BC. However, they raised concerns about technological challenges.

The digital divide (‘the gap between those who have access to information and communication technology and those who do not’ [74]) is a phenomenon that reflects economic, educational, and sociocultural disparities [74]. While internet access has increased globally, older adults access it less than their younger counterparts [70]. In the United States, 25% of those over 65 years of age do not use the internet [75]; in Europe, 51% of those 50 and older do not use the internet [76]. This phenomenon was also called the ‘double burden of exclusion’ during COVID [77]. Stay-at-home directives socially excluded older adults who may not be online, and were therefore also subjected to digital exclusion [77]. While older adults are often positioned as ‘not wanting to engage in newer technology [78], this notion could be shifting. Trends indicate that healthy older adults are increasing their internet use, though the same trend was not observed for those with functional limitations and multiple co-morbidities or the oldest-old [79].

In our study, ACs recounted a range of access ‘pathways’ taken by CTM participants. In some cases, participants struggled to consistently connect to the video conferencing option; some called in (no video) but remained muted; and others opted to attend the outdoor, in-person, distanced meetings instead. During recruitment, many previous participants contacted by our ACs were simply not comfortable with the technology and did not enroll at all—despite a phone-in option.

Our findings highlight the increased need for social connection during the isolating periods of the pandemic—and beyond. Similar to in person CTM programs [42], virtual delivery was able to create a sense of belonging and connectedness. Future studies should seek to mitigate the COVID social connectivity paradox [80] and support online access to physical activity and social connectedness interventions in more vulnerable populations [77]. Using virtual delivery may help us reach a new segment of the older adult population, such as individuals who are homebound (e.g., lacking transport).

In the COVID era other groups successfully engaged older adults through technology-based health promotion interventions. A group of exercise specialists developed a physical activity protocol for live, online group training sessions for older adults (mean 71.5 years old) during COVID quarantines [81]. In this small feasibility study, no adverse events were reported, participant adherence rates were high (90%), and almost all (97%) participants indicated they would partake in a similar online program again in future. Participants were recruited via social media channels, indicating they had some prior digital literacy [81]. Others examined the shift of health promotion programs to the virtual environment during the COVID pandemic, with an emphasis on ensuring access and efficacy for older adults [72]. The Public and Patient Education Department at the Hospital for Special Surgery in New York specializes in programs for those who suffer from or are at-risk of musculoskeletal conditions—90% of whom are 60 years or older. They historically delivered a robust slate of largely in-person workshops, lectures, exercise classes, support groups, and community outreach programs. In a quick pivot, they shifted to offer 79% of their programs through virtual channels in the first 5 months of the pandemic (they did not offer 100% of programs online due to safety concerns). Program reach increased more than 10-fold and participants reported high program satisfaction (90%). Similar to our findings, there were many benefits of virtual programs for participants during lockdown (improved social connectedness, daily habit formation, and positive physical and mental health impact) [72]. Efforts to engage older adults in health promoting technology-based interventions show promise.

In sum, we were able to test a new delivery arm for CTM (virtual) and learned from our providers that it was feasible and acceptable to do so, despite technological challenges. Virtual (or hybrid) interventions may be one effective option moving forward. They improve accessibility for those living in more remote or rural regions where health promotion programs and services might be more limited. Virtual models also broaden the potential to implement at a much larger scope and scale—from one jurisdiction to many jurisdictions should supportive technologies be available.

#### AC adaptations

CTM was designed to meet the needs and address the capacity of different delivery partners in different settings, and to accommodate the interests and capabilities of a wide array of participants [48, 50]. ACs leaned on CTM’s flexibility to adapt components in various ways to support participant needs. We considered one-on-one consultations (where ACs helped design and activate older adults’ activity plans) and group meetings as core components of CTM. However, how these components were delivered (indoors/outdoors) could vary. For example, ACs implemented outdoor, distanced meetings to increase participant responsiveness and feasibility. While participant responsiveness and feasibility are critical to successful program implementation [44, 82], some adaptations might compromise the fidelity of an intervention. For example, ACs who offered weekly instead of biweekly meetings to participants increased the dose of CTM delivered and could conceivably alter (improve) participant-level outcomes. The health impact evaluation (e.g., mobility, physical activity, social health outcomes) of CTM’s virtual delivery is ongoing and will be reported elsewhere.

Adaptation and fidelity exist in a ‘dynamic tension’ in implementation science [83, 84]. One view contends that adaptation is an implementation failure [44, 85, 86]. The opposing view is that adaptation is a *necessary and vital* component of implementing evidence-based interventions. This is particularly true in more uncontrolled settings, when implementing at larger scale and in diverse populations and contexts [84, 87]. Some physical activity studies quantified how different program doses and dose-response relationship influenced older adult health outcomes [88, 89]. However, CTM is a pragmatic intervention, designed with flexibility and scale-up in mind. We balanced these priorities against the need to retain fidelity to what we perceived as core CTM components.

### Strengths

The primary strength of this study lies in the central support unit’s ability to rapidly respond to a pandemic. We leveraged an existing, effective health promoting intervention (CTM) and—with longstanding, well positioned, and committed CTM community delivery partners—rapidly adapted CTM for virtual delivery. Adaptations to CTM were possible as it was designed as a flexible program ‘in the first place’, as opposed to attempting awkward retrofits to less adaptable programs. CTM embraced new (to participants and ACs) audio-visual technology while appreciating its limitations, and we provided participants the training and support required for them to be comfortable ‘users’. CTM also retained other options for older adults to connect (teleconference) which increased accessibility to CTM for older adults across all parts of BC. We aimed to counter, rather than promote ‘social distancing’ [69]. Throughout an ever-evolving public health context, delivery of CTM continued to adapt to government public health restrictions. Finally, virtual delivery of CTM is a format that more readily enables broad scale-up.

### Limitations

We acknowledge several limitations within our study. We conducted a rapid adaptation feasibility study ACs who were all experienced at delivering CTM and had a vested interest in adapting to support older adults under stay-at-home orders during COVID. The presence of members of the central support unit in the interviews may have led ACs to react more positively, and ACs’ general enthusiasm about CTM may have positively skewed our findings. CTM may have felt exclusive; some older adults did not enroll because of technology concerns, despite us offering a telephone (phone-in) option. We did not have time to engage new delivery partner organizations, those with direct access to more isolated and vulnerable older adults (e.g., through home and community care or residential care). Adaptation likely looks significantly different for a more poorly resourced non-profit sector [90].

## Conclusions

Evidence-informed strategies that engage organizations to adapt and deliver virtual health promoting interventions to older adults during what has become a prolonged COVID environment are desperately needed. Programs such as CTM have potential to mitigate mental and physical adverse effects associated with reduced physical activity, social isolation, and loneliness [41, 42]. Adaptation and implementation strategies must be informed by community delivery partners and older adults themselves. Virtual interventions may be an effective option for engaging older adults in health promotion interventions in future and outside of the COVID context (e.g., improved accessibility for those living in more remote or rural regions where health services and health promotion interventions might be more limited). Pragmatic, virtual interventions that can be readily adapted as contexts rapidly shift may forevermore be a part of our changing world.

## Data Availability

The datasets used during the current study are not publicly available as stipulated in our participant consent forms but are available from the corresponding author on reasonable request.

## Abbreviations

AART: Active Aging Research Team
AC: Activity Coach
BC: British Columbia
COVID-19: Coronavirus disease
CTM: Choose to Move
